# Response of Olive Shoots to Salinity Stress Suggests the Involvement of Sulfur Metabolism

**DOI:** 10.3390/plants10020350

**Published:** 2021-02-12

**Authors:** Muhammad Ajmal Bashir, Cristian Silvestri, Eleonora Coppa, Elena Brunori, Valerio Cristofori, Eddo Rugini, Touqeer Ahmad, Ishfaq Ahmad Hafiz, Nadeem Akhtar Abbasi, Muhammad Kausar Nawaz Shah, Stefania Astolfi

**Affiliations:** 1 Department of Horticulture, PMAS Arid Agriculture University, Rawalpindi 46300, Pakistan; muhammadajmal@unitus.it (M.A.B.); touqeer@uaar.edu.pk (T.A.); drishfaq@uaar.edu.pk (I.A.H.); nadeem.abbasi@uaar.edu.pk (N.A.A.); 2 Department of Agriculture and Forest Sciences (DAFNE), University of Tuscia, 01100 Viterbo, Italy; eleonoracoppa@libero.it (E.C.); valerio75@unitus.it (V.C.); rugini@unitus.it (E.R.); 3 Department for Innovation in Biological, Agro-Food and Forest Systems, Tuscia University, via San Camillo de Lellis snc, 01100 Viterbo, Italy; brunori@unitus.it; 4 Department of Plant Breeding and Genetics, PMAS Arid Agriculture University, Rawalpindi 46000, Pakistan; knshah@uaar.edu.pk

**Keywords:** *Olea europaea* L., osmotin, salinity, sulfur assimilation, transgenesis

## Abstract

Global warming has two dangerous global consequences for agriculture: drought, due to water scarcity, and salinization, due to the prolonged use of water containing high concentrations of salts. Since the global climate is projected to continue to change over this century and beyond, choosing salt-tolerant plants could represent a potential paramount last resort for exploiting the secondary saline soils. Olive is considered moderately resistant to soil salinity as compared to other fruit trees, and in the present study, we investigated the influence of NaCl solutions (ranging from 0 to 200 mM) in a salt-tolerant (cv Canino) and two of its transgenic lines (Canino AT17-1 and Canino AT17-2), overexpressing tobacco osmotin gene, and in a salt-sensitive (Sirole) olive cultivar. After four weeks, most of the shoots of both Canino and Sirole plants showed stunted growth and ultimate leaf drop by exposure to salt-enriched media, contrary to transgenic lines, that did not show injuries and exhibited a normal growth rate. Malondialdehyde (MDA) content was also measured as an indicator of the lipid peroxidation level. To evaluate the role of the S assimilatory pathway in alleviating the adverse effects of salt stress, thiols levels as well as extractable activities of ATP sulfurylase (ATPS) and O-acetyl serine(thiol)lyase (OASTL), the first and the last enzyme of the S assimilation pathway, respectively, have been estimated. The results have clearly depicted that both transgenic lines overexpressing osmotin gene coped with increasing levels of NaCl by the induction of S metabolism, and particularly increase in OASTL activity closely paralleled changes of NaCl concentration. Linear correlation between salt stress and OASTL activity provides evidence that the S assimilation pathway plays a key role in adaptive response of olive plants under salt stress conditions.

## 1. Introduction

Global warming has two dangerous global consequences for agriculture: drought, due to water scarcity, and salinization, due to the prolonged use of water containing high concentrations of salts. Thus, it is important to identify plants that can cope with drought and salt stress to increase crop resilience under different climatic changes. Olive (*Olea europaea* L.) tree belonging to family Oleaceae, is one of the most popular species of the genus *Olea* which is used for food purpose [1,2]. Olive is widely grown all over the world, with a great potential for expansion because of its ability to cope with unfavorable conditions [3,4,5]. However, most of the olive production (about 98%) comes from the Mediterranean basin [1]. Therefore, climate change will have a strong impact on olive cultivation as this area is warming up to 20% faster than the rest of the world average.

Olive is known as a glycophytic species presenting medium tolerance to salts [6] with marked differences among cultivars [7,8,9]. One of the major effects of salt stress is oxidative stress, resulting in increased levels of reactive oxygen species (ROS) [10]. ROS have the potential to interfere with many cellular components, causing cell membrane and other cellular structures damage, metabolic disorders, inhibited photosynthesis, and impaired nutrient uptake [11]. To face increased ROS production, plants have developed defensive strategies, including enzymatic and non-enzymatic antioxidant systems. Thiol compounds, and in particular glutathione (GSH) [12], act as antioxidants that protect plants against the damaging effects of increased ROS levels under salt stress conditions [13]. Furthermore, plant responses to salinity include the synthesis of harmonious osmolytes, and stimulation of phytohormones production [14]. The molecular mechanism involves the overexpression of particular genes triggering different stress-related proteins and play a pivotal role in stress adaptation mechanisms [15].

Among the several stress-related proteins, osmotin is known to be induced in response to both biotic and abiotic stresses in plants [16]. Osmotin is a cationic protein belonging to the PR5 family proteins [17,18]. It has been suggested that osmotin confers the tolerance to salinity stress by increasing the accumulation of osmolytes, such as proline and glycine betaine [15,17,19,20].

The main objective of this study was to understand whether osmotin is involved or not, to show resistance against salt stress in olive shoots. This goal was achieved by using a salt-tolerant (cv Canino), with two of its transgenic lines (Canino AT17-1 and Canino AT17-2), overexpressing tobacco osmotin gene, and a salt-sensitive (cv Sirole) olive cultivar, exposed to NaCl solutions (ranging from 0 to 200 mM). We investigated whether the mechanisms of proline accumulation and lipid peroxidation (as an estimate of oxidative damage) were affected by the presence of the osmotin gene.

Furthermore, as GSH plays an active role in the process of response to salinity, we tested the hypothesis that a close relationship might exist between salt tolerance and sulfur (S) assimilation rate. Thus, changes in thiols levels as well as in extractable activities of ATP sulfurylase (ATPS) and *O*-acetyl serine(thiol)lyase (OASTL), the first and the last enzyme of the S assimilation pathway, respectively, have been estimated in plants exposed to salt stress.

## 2. Results

### 2.1. Effect of NaCl on In Vitro Shoot Growth Performance

Statistical analysis showed significant interaction between relative growth rate at different concentrations of NaCl for both wt genotypes (Canino and Sirole) and transgenic lines (Canino AT17-1 and Canino AT17-2). Positive interaction between each genotype and treatment with 50 mM NaCl has been observed, as showed in Table 1.

**Figure 1:**
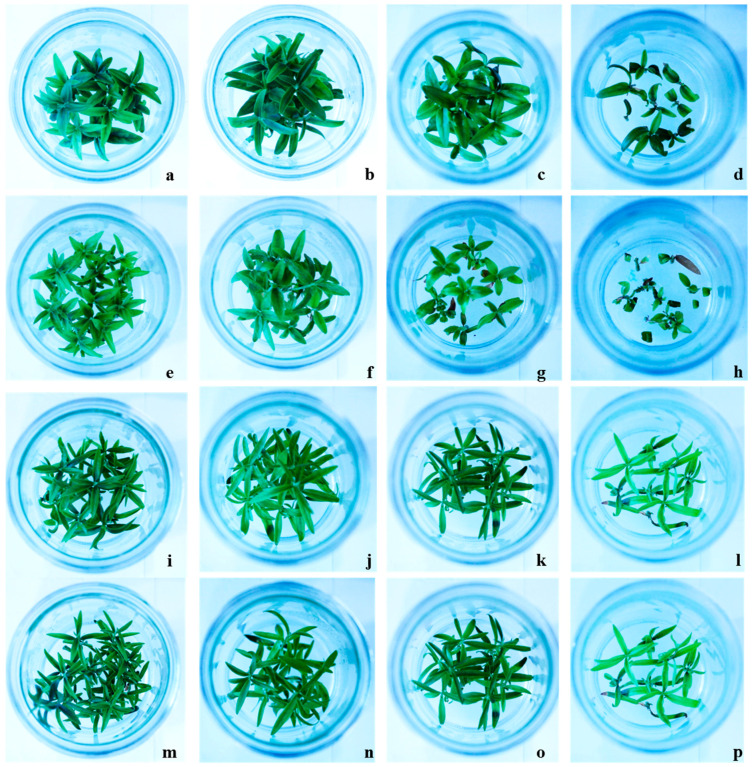
Effect of different concentrations of NaCl on different olive cultivars Canino (**a**–**d**) at 0, 50, 100, and 200 mM NaCl, respectively, Sirole (**e**–**h**) at 0, 50, 100, and 200 mM NaCl, respectively, along with two transgenic Canino AT17-1 (**i**–**l**) at 0, 50, 100, and 200 mM NaCl, respectively and Canino AT17-2 (**m**–**p**) at 0, 50, 100, and 200 mM NaCl, respectively.

The in vitro shoot growth was significantly influenced by NaCl concentration in the growing media (Figure 1). The highest relative growth rate (RGR) has been observed in Canino shoots in medium enriched with 50 mM NaCl, followed by Sirole at the same salt concentration. However, both cultivars showed a significantly reduced RGR at higher NaCl concentrations (100 and 200 mM), compared to the transgenic lines, Canino AT17-1 and Canino AT17-2, which showed a RGR of 3.92 and 3.72 at 100 mM NaCl, and 2.55 and 1.77 at 200 mM NaCl, respectively (Table 1).

The McKinney index (MKI) and tolerance index (TI) were also calculated to evaluate the level of shoot chlorosis and necrosis resulting from NaCl treatment. Our results showed that MKI was positively correlated with increasing NaCl concentrations. However, values of MKI were higher in Canino and Sirole shoots, as compared to Canino AT17-1 and Canino AT17-2 transgenic lines (Table 2).

Contrarily, the exposure of shoots to increasing NaCl concentrations resulted in values for TI of 4.7, 16.5, and 23.4% for Canino, and 9.1, 18.2, and 20.5% for Sirole, at 50, 100, and 200 mM, respectively (Table 2). Interestingly, both transgenic lines (Canino AT17-1 and Canino AT17-2) responded better to exogenous NaCl treatments, showing values for TI of 28.8, 46.4, and 47.9% and 14.3, 25.9, and 32.30%, at 50, 100, and 200 mM NaCl, respectively.

### 2.2. Effect of Different Levels of NaCl on Photosynthetic Pigments

**Figure 2:**
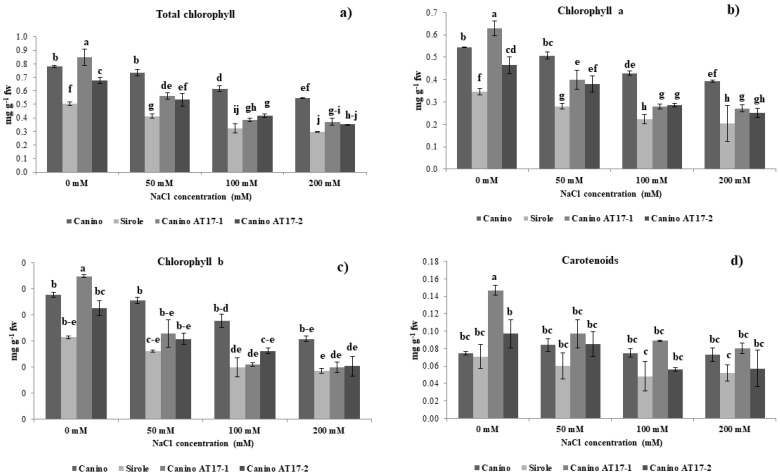
(**a**) Total chlorophyll content, (**b**) chlorophyll a, (**c**) chlorophyll b, and (**d**) carotenoids of olive cv Canino, Sirole, and the transgenic lines Canino AT17-1 and Canino AT17-2 under different concentration of NaCl. Data are means ± SD of four independent replications run in triplicate. Significant differences between samples are indicated by different letters according to Fisher’s test (*p* < 0.01).

After four weeks of exposure to salt stress, all genotypes showed typical toxicity symptoms, such as decreased chlorophyll content. Total chlorophyll concentration of Canino shoots slowly decreased with increasing NaCl concentration (7, 22, and 30%, at 50, 100, and 200 mM, respectively). While on the other hand, Sirole and both transgenic lines exhibited relatively worse response to salt stress, resulting in more rapid and greater reduction of total chlorophyll concentration. The highest NaCl concentration (200 mM) resulted in 40, 57, and 48% lower chlorophyll content in Sirole, Canino AT17-1 and Canino AT17-2, respectively (Figure 2a–c).

Carotenoid concentration was approximately the same in both Canino and Sirole shoots in control condition (0 mM NaCl), whereas both transgenic lines showed higher carotenoid content. In particular, Canino AT17-1 showed an almost twofold increase from that of Canino wt, whereas in Canino AT17-2, it increased by a lesser extent (30% with respect to Canino wt). Furthermore, Canino wt maintained approximately the same carotenoid concentration irrespective of the NaCl in the growth medium, in Sirole and in both transgenic lines, it significantly declined with increasing NaCl concentration. However, when the growth medium was supplemented with 200 mM NaCl, the highest values for carotenoid concentration were found in Canino AT17-1, showing values similar to Canino wt, followed by Canino AT17-2 (Figure 2d).

### 2.3. Effect of Different Levels of NaCl on Malondialdehyde (MDA) Content

**Figure 3:**
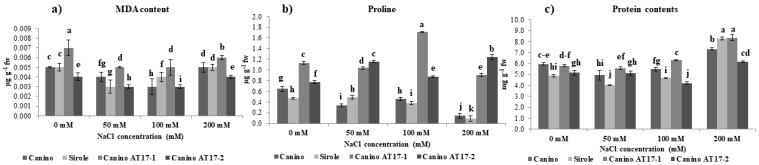
(**a**) Malondialdehyde, (**b**) proline content and (**c**) protein content of olive cv Canino, Sirole, and the transgenic lines Canino AT17-1 and Canino AT17-2 under different concentration of NaCl. Statistics as in Figure 2.

Oxidative stress due to the presence of NaCl in the growth medium often results in lipid peroxidation which is generally evaluated by enhanced MDA content. However, our results showed that salt exposure did not increase shoot MDA levels of both wt and transgenic lines irrespective of NaCl concentrations (Figure 3a). On the other hand, it is interesting to note that the transgenic line AT17-1 showed higher MDA concentration (+30%) as compared to Canino wt under control conditions (0 mM NaCl).

### 2.4. Effect of Different Levels of NaCl on Proline Accumulation

The proline accumulation in the salt-tolerant cv Canino was significantly higher (36%) as compared to the salt-sensitive one (cv Sirole), but the same metabolite accumulated in even greater extent in the shoots of Canino AT17-1 and Canino AT17-2 (+75 and +20% compared to Canino wt in the control condition, respectively). In addition, both Canino and Sirole shoots were severely affected by 200 mM NaCl treatment and showed a significative decrease of proline concentration (78 and 82%, respectively) (Figure 3b). whereas, both transgenic lines, resulted a consistent accumulation of proline in shoots when exposed to increasing NaCl concentrations. It is noteworthy that the increase of proline accumulation due to highest salt treatment (200 mM) in both Canino AT17-1 and Canino AT17-2 reached levels almost 7- and 9-fold higher, respectively, as compared to those found in Canino wt in the same condition (Figure 3b).

### 2.5. Effect of Different Levels of NaCl on Protein Contents

Data indicated that imposed NaCl stress was closely related to shoot protein concentration as showed in Figure 3c. There was a lower accumulation of protein in shoots of all genotypes as well under control conditions (without NaCl). Furthermore, a significant increase in protein accumulation was observed in all tested shoots of both wt and transgenic lines but no significant differences were observed between wt cultivars and transgenic lines overexpressing osmotin gene at higher concentrations of NaCl (200 mM).

### 2.6. Effect of Different Levels of NaCl on Non-Protein Thiol Contents

**Figure 4:**
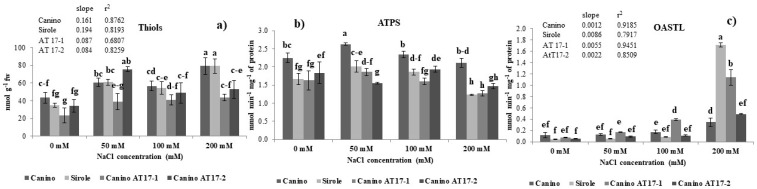
(**a**) Thiols content, (**b**) ATP sulfurylase (ATPS) and (**c**) O-acetyl serine(thiol)lyase (OASTL)activities of olive cv Canino, Sirole, and the transgenic lines Canino AT17-1 and Canino AT17-2 under different concentration of NaCl. Statistics as in Figure 2.

It is well known that thiol compounds, and GSH particularly, act as antioxidants to counteract oxidative stress induced by NaCl toxicity [21]. Our results show that non-protein thiols production and NaCl concentration in the growth medium significantly correlated in all four studied genotypes (Canino r^2^ = 0.8762; Sirole r^2^ = 0.8193; Canino AT17-1 r^2^ = 0.6807; Canino AT17-2 r^2^ = 0.8259), even if at different extent) (Figure 4a). As evidenced by the slope of the increase of thiols accumulation with increasing salt concentration, this response showed the following trend: Sirole > Canino > Canino AT17-1 = Canino At17-2) (Figure 4a).

### 2.7. Effect of Different Levels of NaCl on ATPS and OASTL Activities

In order to evaluate if changes in thiols level required an adjustment in plant S assimilation rate, the changes in ATPS and OASTL activity, the first and last enzyme of the sulfur assimilation pathway, respectively, were measured (Figure 4b,c). A slight induction of ATPS activity was observed in response to the lowest NaCl concentration (50 mM) in Canino and Sirole cultivars, and in transgenic line Canino AT17-1, but the increase ranged between 15 and 20% (Figure 4b). On the other hand, the same stress condition resulted in slightly decreased ATPS activity (−15%) in Canino AT17-2 (Figure 4b). No significant differences were observed in ATPS activity at 100 mM NaCl, relative to controls, in all four genotypes and, finally, when plants were treated with the highest salt concentration (200 mM) ATPS activity levels significantly decreased compared to control (decrease ranged from 25 to 30%), except for Canino which maintained approximately the same activity as in the control condition (Figure 4b).

As for thiols, OASTL activity was positively correlated with salt concentration in the growth medium (Figure 4c) and, indeed, the correlation was very high in all four studied genotypes (Canino r^2^ = 0.9185; Sirole r^2^ = 0.7917; Canino AT17-1 r^2^ = 0.9451; Canino AT17-2 r^2^ = 0.8509) (Figure 4c). However, distinct differences among genotypes were observed and, as evidenced by the slope of the increase of OASTL activity with increasing salt concentration, the induction showed the following trend: Sirole > Canino AT17-1 > Canino At17-2 > Canino) (Figure 4c).

### 2.8. Principal Component Analysis (PCA)

To test the sampling adequacy for the data set to multivariate statistical analysis (such as PCA) the Kaiser–Meyer–Olkin (KMO) measure has been used for the overall data set and for each individual variable. KMO statistic varies between 0 and 1, Hair et al. [22] recommends accepting values of 0.5 or more, appropriate as regards to minimum threshold for robustness of factor structure. Findings (KMO = 0.509) stressed the need for a data reduction, to avoid redundancy due to high correlation from variables as also showed by Bartlett’s test. Variables selected showed individual KMO greater than 0.5—not including TI, Ch ToT, carotenoids, ATPS. The KMO and Bartlett’s test performed for the new dataset justified the multivariate analysis (PCA) (Table 3). Pearson’s correlation matrix (Table 4) was detected and significant correlations displayed in bold.

**Figure 5:**
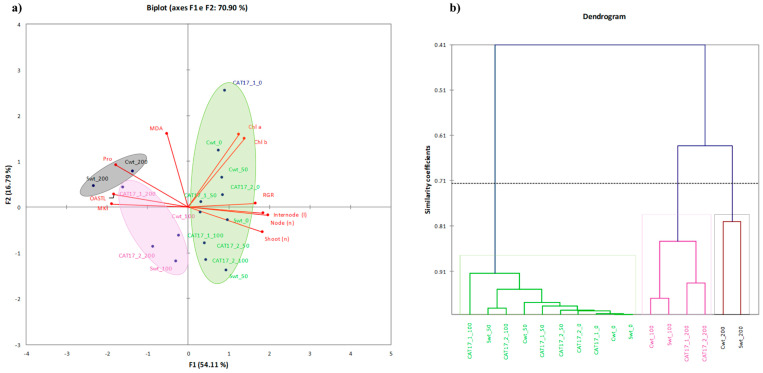
PCA representation of the factors levels on the plane spanned by the principal axes F1 and F2 (**a**) and dendrogram of cluster analysis (**b**). Legend: Swt-0—Sirole wt 0mM NaCl, Swt-50—Sirole wt 50 mM NaCl, Swt-100—Sirole wt 100 mM NaCl, Swt-200—Sirole wt 200 mM NaCl; Cwt-0—Canino wt 0 mM NaCl, Cwt-50—Canino wt 50 mM NaCl, Cwt-100—Canino wt 100 mM NaCl, Cwt-200—Canino wt 200 mM NaCl; CAT17-2-0—Canino AT17-2 0 mM NaCl, CAT17-2-50—Canino AT17-2 50 mM NaCl, CAT17-1-100—Canino AT17-1 100 mM NaCl, CAT17-1-200—Canino AT17-1-200 mM NaC; CAT17-2-0—Canino AT17-2-0 mM NaCl, CAT17-2-50—Canino AT17-2-50 mM NaCl, CAT17-2-100—Canino AT17-2-100 mM NaCl, CAT17-2-200—Canino AT17-2-200 mM NaCl.

Principal component analysis (PCA) was carried out using the selection of factors loading and scores showed in Figure 5a and dendrogram of cluster analysis in Figure 5b. In Table 5, the set of the eigenvalues of PCA was reported, with the amount of inertia explained by each corresponding axis (F), and the cumulate inertia. Two principal axes (F1 and F2) were selected, which explained about 71% of the total variation (measured by the inertia), yet 60% is the minimum cumulate quality of representation of all the variables. The contribution of variables to the first principal axis (F1) (also shown on the principal plane spanned by F1 and F2 in Figure 5 is due mostly to the RGR, node and shoot number, internode length, MKI, protein, and OASTL contents. The second principal axis (F2) mainly contributes Chl a and b content and MDA.

## 3. Discussion

Owing to their sessile lifestyle, plants are continuously challenged with a broad range of environmental stresses, among which salinity stress is recognized as one of the most negatively impacting stress on plant growth and crop yield [23]. Plant response to stress commonly rely on expression of specific genes and synthesis of a large number of stress-related proteins, which plays a crucial role in stress adaptation [24]. Thus, the expression of genes involved in signaling and regulatory pathways could represent one of the most promising approach for improving stress tolerance in plants. In the past two decades, overexpression of osmotin gene in tobacco [18,25], tomato [26], strawberry [20,27], and chili pepper [24] have been reported to enhance tolerance under NaCl-mediated salinity stress.

Olive (*Olea europaea* L.) is a typical crop of the Mediterranean basin, where soil salinization is projected to worsen because of global climate change. In this study, the contribution of osmotin has been investigated in olive in response to salinity by using four different olive genotypes: a salt-tolerant (cv Canino) with its two transgenic lines (Canino AT17-1 and Canino AT17-2), overexpressing tobacco osmotin gene, and a salt-sensitive (cv Sirole). Since one of the major effects of salt stress is oxidative stress, resulting in increased levels of reactive oxygen species (ROS) [10], and being GSH actively involved as antioxidant in the process of response to salinity, we also tested the hypothesis that a close relationship might exist between salt tolerance and S assimilation rate.

The evaluation of olive resistance to salinity stress in terms of the McKinney index (MKI) and tolerance index (TI), indicating the level of shoot chlorosis and necrosis, respectively, we demonstrated that the lower effects of salt-induced stress were observed in both transgenic lines (Table 2). Zero MKI values, corresponding to green leaves and stems, were observed in all tested genotypes grown in control condition (0 mM NaCl) until the end of the experiment. Both Canino and Sirole showed significantly increased chlorosis, with increasing salt concentration in the growth medium, while the transgenic lines showed normal growth even at highest salt stress level (Figure 1). This is a clear indication that both transgenic lines responded better to exogenous NaCl treatments, showing lower MKI values as well as higher TI values, than Canino and Sirole (Table 2). These findings are consistent with previous study showing that *Myrtus communis* L. plants exposed to 150 and 250 mM NaCl showed high chlorosis levels, with highest MKI values found in shoots exposed to 250 mM NaCl for 30 days [28].

Nevertheless, this effect seemed not to be directly linked to total chlorophyll content. In fact, in our experimental conditions, we observed that the treatment with NaCl was more negatively impacting on Sirole and both transgenic lines, which showed a higher rate of chlorophyll loss from shoots, as compared to Canino (Figure 2). In fact, in this latter case concentration of chlorophyll remained relatively stable from 0 to 50 mM NaCl, to later decrease further increasing NaCl concentration (Figure 2). Salinity effect on chlorophyll content is often ambiguous. For instance, it has been recently demonstrated that salt stress increased photosynthetic pigments in both tolerant and sensitive alfalfa ecotypes [29]. This could also be the case for olive, which has not been previously reported and suggests that chlorophyll content alone do not to provide an indication of the extent of salt stress.

It is well known that several abiotic stresses influence the accumulation of carotenoids, whose regulation is suggested to lead to stress tolerant phenotypes. In our experimental conditions, we found that both transgenic lines showed higher carotenoid contents as compared to Canino and Sirole in control condition (0 mM NaCl), whereas Sirole and transgenic lines exhibited a net reduction of carotenoids level under salt stress (Figure 2d). This data was in consistent with those results obtained by [30], which showed that β-carotene content decreases in pepper under oxidative-induced stress.

Our findings under salt stress resulted in similar decrease rate of carotenoids for genotypes with contrasted tolerance (Sirole and Canino transgenic lines), suggesting that carotenoids metabolism is not directly involved in the stress tolerance mechanism, and changes in carotenoid accumulation are rather due to plant general metabolic modifications occurring during adaptation to stress conditions.

Although it has not been clearly demonstrated that the mechanism by which osmotin improves plant response under abiotic stress, a few studies have shown that under salinity stress osmotin allows the reduction of Na^+^ ions uptake into the cytoplasm though a Na^+^/H^+^ anti-porter [31], in addition that osmotin could be involved in osmotic adjustment of plants under stress either by facilitating the accumulation or compartmentation of different solutes in intracellular spaces [18]. As salinity results in water stress, plants have to accomplish an osmotic adjustment as well to maintain their metabolic activities. Osmotic adjustment is achieved by the accumulation of the compatible solutes such as free proline. It has been reported that stress tolerance increases with increasing proline accumulation [32] and, furthermore, that proline can induce the expression of several genes involved in stress tolerance [33], suggesting a role for this metabolite not only to low water potential but also in plant protection from osmotic stress [34]. Thus, the transgenic approach could represent a promising strategy to increase proline production and improve stress tolerance in plants.

Interestingly, our results showed that the salt-tolerant cv Canino accumulated more proline as compared to the salt-sensitive one (cv Sirole) (Figure 3b), confirming the previous study which suggested that proline accumulation is genotype-dependent [35]. Moreover, we found that shoots of both transgenic lines were able to increase proline accumulation with increasing NaCl concentrations in the medium and when they were exposed to the highest salt concentration (200 mM), proline content accumulated substantially (almost 7- and 9-fold higher in Canino AT17-1 and Canino AT17-2, respectively, compared to those found in Canino wt in the same condition) (Figure 3b). This result reasonably suggests that the presence of osmotin gene could play an important role in the regulation of the mechanism of proline accumulation under salt stress. Proline synthesis is commonly induced under stressed conditions through an intercellular signal pathway mediated by ROS accumulation [34]. Certainly, in plants exposed to environmental constraints, the accumulation of ROS is generally associated to proline accumulation [34].

Salt stress provokes an excessive ROS production resulting in oxidative damage leading to cell injury and death [36]. Therefore, we compared the changes in MDA concentration (as an estimate of lipid peroxidation) to assess the degree of oxidative damage induced by NaCl treatment [37]. Our data showed that all tested genotypes were able to maintain on average a lower MDA content than that found in control condition (Figure 3a). This result might be explained by assuming a significant role of proline in modulating both ROS generation and scavenging of ions [38], but also to high efficiency of the antioxidant system leading to an increased stress tolerance. This result meant that the olive shoots had a proper membrane stability even under salt stress.

According to the literature, plants possess some strategies to cope with oxidative stress, such as enzymatic and non-enzymatic antioxidant systems. Non-enzymatic systems include thiol compounds such as glutathione and cysteine, playing an important role in improving the tolerance to oxidative stress induced by NaCl toxicity [13,34,39]. Our results showed that non-protein thiols production and NaCl concentration in the growth medium were significantly positively correlated in all four studied genotypes (Canino r^2^ = 0.8762; Sirole r^2^ = 0.8193; Canino AT17-1 r^2^ = 0.6807; Canino AT17-2 r^2^ = 0.8259) (Figure 4a) and Sirole cv exhibited the highest slope of the increase of thiols accumulation with increasing salt concentration (Figure 4a). Thus, based on our results, we suggest that in Sirole, being a salt-sensitive cv, a large amount of ROS is likely produced due to salt stress, leading to increased pressure on ROS scavenging activity and thus to accumulate increased amounts of antioxidant compounds to cope with stress condition. Moreover, this response was well in line with the lowest proline contents found in Sirole.

Changes in thiols production reasonably require an adjustment of sulfate assimilation rate under salt stress, thus the activities of ATPS and OASTL, the first and the last enzyme of S assimilation pathway, respectively, have been estimated in plants exposed to salt stress. In this study, the increase of thiols production (Figure 4a) was parallel with that of OASTL activity (Figure 4c), indicating that S assimilation pathway plays an important role in the regulation of plant response to salt stress. As for thiols, OASTL activity increased with increasing salt concentration in the growth medium and, indeed, the correlation was very high in all four studied genotypes (Canino r^2^ = 0.9185; Sirole r^2^ = 0.7917; Canino AT17-1 r^2^ = 0.9451; Canino AT17-2 r^2^ = 0.8509) (Figure 4f), and as for thiols, the highest slope of the linear correlation was found in Sirole cv (Figure 4c). Interestingly, for both thiols and OASTL activity the lowest slope of the linear correlation was found in both transgenic lines (Figure 4a,c). The different pattern observed for ATPS and OASTL activity could be explained by assuming that OASTL directly catalyzes the biosynthesis of cysteine [40], the precursor for glutathione synthesis, and thus its activity could play a more important role in adaptation mechanisms to salt stress condition, than ATPS (Figure 4b). These results are well in line with previous studies showing ATPS activity increase [41,42] as well as decrease [43] in response to salt treatment.

Summarizing all these findings by the PCA analysis computed with the complete dataset comprising MDA and thiols levels, and enzyme activities (Figure 5) appeared a clear clustering along the first component (describing 54.11% of the total variance) based on NaCl concentration in the media. Most of samples treated with the highest salt concentrations were found on the negative axis and interestingly with a negative loading for MDA, proline, and OASTL (Figure 5). In conclusion, our results suggested that both Canino AT17-1 and Canino AT17-2 transgenic lines had better salt tolerance ability as compared to their relative wt (Canino), suggesting that the presence of osmotin could likely improve olive salt stress tolerance. Although the mechanism involved is still not fully understood, our results indicate that osmotin could play an important role in the regulation of proline accumulation, which in turn would exert a protective role preventing or modulating ROS generation.

Furthermore, the data obtained suggests an interesting interaction between salt stress and S metabolism and the involvement of this nutrient during adaptation of olive shoots to salt stress. Indeed, we have revealed that antioxidant response to scavenging excessive ROS requires the stimulation of S assimilation rate to sustain higher demand of reduced S.

## 4. Materials and Methods

### 4.1. Plant Material and Growing Conditions

Shoot culture derived from in vitro propagation of two olive cultivar (Canino and Sirole) and two transgenic lines (Canino AT17-1 and Canino AT17-2) expressing tobacco osmotin gene obtained by A. tumefaciens-mediated transformation of olive somatic embryos [3,44,45] have been used.

The shoots were sub-cultured for four weeks on OM medium [46] containing mannitol 36 g/L, L-glutamine 2.2 g/L, and zeatin 1 mg/L (added filter-sterilized after autoclave) and plant agar (Duchefa, NL) 0.6%. The pH of the medium was adjusted to 5.8 before autoclaving. Three 250 mL jars each containing ten single node explants were sub-cultured in with 50 mL proliferation medium as above described, supplemented with three different NaCl concentrations NaCl (50, 100 and 200 mM), corresponding to the electrical conductivity reported in Table 6, compared to the control (NaCl 0 mM). Explants were kept under controlled conditions in a growth chamber with a day/night cycle of 16/8 h at 24 ± 1 °C air temperature, 80% relative humidity, and 40 mmol m^−2^ s^−1^ light intensity. Data collection were performed after 28 days in culture.

**Figure 6:**
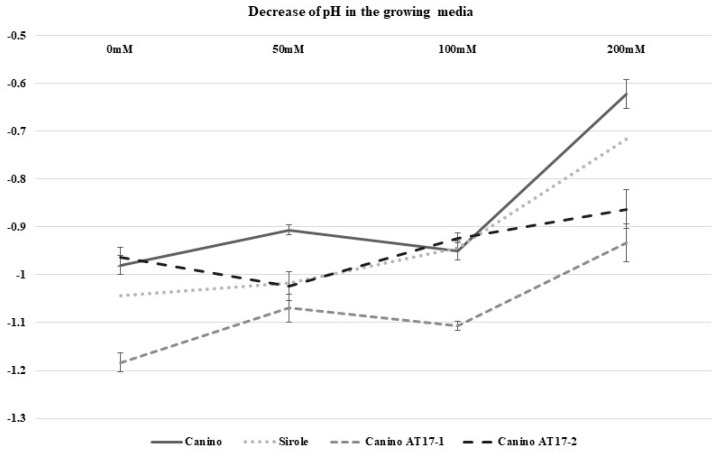
Media pH decrease after 4 weeks of culture of the 4 genotypes maintained on media enriched with different NaCl concentrations.

Sampling for both morphological observations and analyses was performed after 30 days and decrease in medium pH was calculated as well (Figure 6). Samples collected for analyses were stored at −80 °C until use.

### 4.2. Test for Transgenesis

Before starting the present experiment, the shoots of transgenic olive lines were tested again for kanamycin resistance, at 150 mg L^−1^ kanamycin-enriched media, and the presence of the transgenic genes in their DNA were ascertain. Olive genomic DNA was extracted from young leaves (150m g of fresh tissue) using a based CTAB (cetyl-trimethyl-ammonium bromide) method [47]. DNA concentration and quality were determined by 1% agarose gel electrophoresis and using a Nanodrop Bioanalyzer ND1000 (Thermo Scientific). Then, the transformant was screened for the transgene by polymerase chain reaction (PCR) using the osmotin-specific primers Osm-F (5′-CCAACAACCCAACTTGTTAAAA-3′) and Osm-R (5′-CGACAGAATAATTTGACCAAAAG-3′).

**Figure 7:**
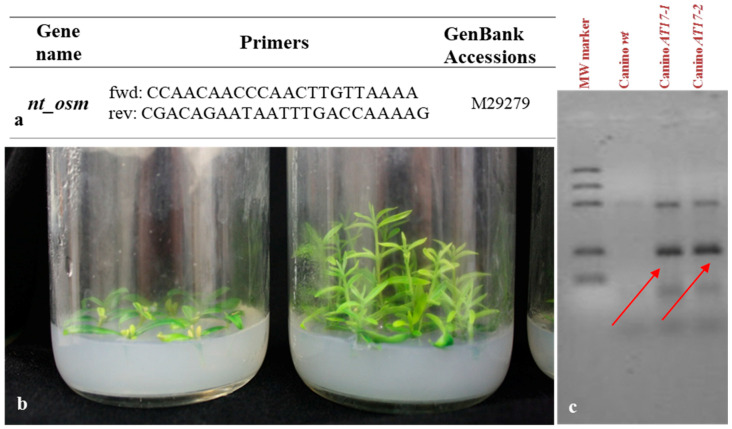
Tobacco osmotin gene with GenBank accession number and associated primers utilized in PCR (**a**), Canino wt (Control) and transgenic Canino AT17-1 growing on selection medium enriched with kanamycin (150 mg L^−1^) (**b**), Analysis of tobacco osmotin gene integration in olive transgenic shoots: red arrows indicate the PCR products of the transgenic clones Canino AT17-1 and Canino AT17-2 (**c**).

The PCR conditions were 95 °C for 5 min; 35 cycles of 94 °C for 30 s, 60 °C for 45 s, and 72 °C for 80 s, and final extension at 72 °C for 10 min. The reaction products were separated electrophoretically on 1.5% (*w*/*v*) agarose gel, stained with ethidium bromide, and photographed with a digital camera (Nikon coolpix 5700) (Figure 7).

### 4.3. Growth Analysis

To evaluate shoot proliferation, the data for node number, shoot number, shoot length (cm), and mean internode length (cm) were collected. The relative growth rate (RGR) index was also calculated after four weeks of culture as per methods described by [48].

McKinney index (MKI) and tolerance index were calculated to evaluate the damage caused by salinity stress and data were recorded based on a rating scale by dividing each shoot into ten classes using MKI (Table 7), as reported by [28]. The estimation of the tolerance index (TI) was performed based on chlorophyll content by using the formula described by [49].

### 4.4. Photosynthetic Pigments

To determine pigment concentration, 100 mg of fresh leaves were collected in 15 mL tubes and 4 mL of methanol 100% were added for the extraction. The tubes were heated at 65 °C for 10 min and then stored at 4 °C for 24 h. The values of total chlorophyll content, chlorophyll a, chlorophyll b, and carotenoid content were determined after centrifugation at 5000 rpm for 5 min, using a spectrophotometer (model EVO 60, made: Thermo Fisher Scientific Inc.) by following the methods described by [50].

### 4.5. Proline Accumulation

Freshly harvested leaf samples (100 mg of fresh weight) were collected, and proline concentration was determined colorimetrically, based on proline reaction with ninhydrin according to [51]. Briefly, shoot tissues (0.2 g) were powdered in liquid nitrogen, and then homogenized in a 1:1:1 solution of proline, ninhydrin, and glacial acetic acid. After an incubation at 100 °C for 1 h, the reaction was arrested in an iced bath. The red product (chromophore) was extracted with 4 mL toluene and the absorbance at 520 nm was determined with a spectrophotometer EVO 60 (Thermo Fischer Scientific Inc. Waltham, Massachusetts, United States). Free proline concentration was calculated using a standard curve.

### 4.6. Determination of MDA

The lipid peroxidation was estimated as malondialdehyde (MDA) content by following the methods described by [52]. Briefly, fresh shoot tissues (0.2 g) were homogenized in 10 mL of 0.25% TBA buffer prepared in 10% TCA, crushed with a mortar and pestle. Extracts were then heated at 95 °C for 30 min and then quickly cooled in ice. After a centrifugation step at 10,000 rpm for 10 min, the absorbance of the supernatant was measured at 532 nm absorbance in spectrophotometer EVO 60 (Thermo Fischer Scientific Inc. Waltham, Massachusetts, United States). The correction of non-specific turbidity was done by subtracting the absorbance values taken at 600 nm absorbance. The level of lipid peroxidation was presented as mmol g^−1^ fresh weight by using coefficient of 155 mM cm^−1^.

### 4.7. Non-Protein Thiols Extraction and Determination

Water soluble non-protein thiol compounds were determined colorimetrically with 5,5′ dithio-bis-(2-nitrobenzoic acid) (DTNB), following the procedure reported by [53]. Briefly, both shoots and roots (1 g fresh weight) were grounded in liquid N_2_ and extracted in 3 mL of a solution composed of 80 mM trichloroacetic acid (TCA), 1 mM ethylenediaminetetraacetic acid (EDTA), 0.15% (*w*/*v*) ascorbic acid, and 10% (*w*/*v*) polyvinylpolypyrrolidone (PVP). The final pH was between 5 and 6. After a centrifugation step (30 min at 4000 g and 4 °C), the supernatants were collected, and the concentrations of DTNB-reactive compounds were measured spectrophotometrically by reading the A415. Measurements of extracts were corrected for the absorbance at 415 nm in both the absence of DTNB (cuvette with extract but no DTNB) and the basal absorbance of DTNB (cuvette with DTNB but no extract or standard).

### 4.8. Determination of ATPs and OASTL

Shoot tissues (1 g fresh weight) were powdered in a pre-chilled mortar with liquid N_2_. Cold extraction buffer, containing 50 mM HEPES-KOH (pH 7.4), 5 mM MgCl_2_, 1 mM EDTA, 10% (*v*/*v*) glycerol, 0.1% (*v*/*v*) Triton X-100, 5 mM dithiothreitol (DTT), 1 mM phenylmethylsulfonyl fluoride (PMSF), and 1% (*w*/*v*) polyvinylpyrrolidone (PVP), was added in a ratio of 1:7 (*w*/*v*). The following extraction steps were performed according to the method described by [53]. The activity of ATP sulfurylase (ATPS; EC 2.7.7.4) was determined by the bioluminescence technique according to [53]. The ATP production during the enzyme reaction is coupled to the light producing reaction catalyzed by firefly luciferase (E.C. 1.13.12.7). The reaction mixture contained in a total volume of 0.25 mL:16 mM tris-acetate buffer pH 7.75, 8 mM APS, 68 mM Na_4_P_2_O_7_, 40 mL of firefly luciferase (ATP Monitoring Reagent, ThermoLabSystems), and 5 mL of sample. Light emission was measured with LKB 1250 luminometer. *O*-acetylserine (thiol) lyase (OASTL; EC 4.2.99.8) activity was determined colorimetrically, measuring the cysteine synthesis using ninhydrin as described by [53]. The crude extract was added to a final volume of 0.9 mL of reaction mixture, containing 30 mM of K-phosphate buffer (pH 7.5), and 12 mM of OAS. The reaction was started by the addition of 6 mM Na_2_S, and after 10 min at 90 °C, it was stopped by the addition 50 µL of a 20% (*v*/*v*) trichloroacetic acid solution and vigorous stirring.

### 4.9. Estimation of Protein Content

Protein concentration in the extracts was determined by a standard spectrophotometer (595 nm) following the procedures described by Bradford [54], using the Pierce™ Coomassie Plus™ Protein Assay Reagent (Thermo Fisher Scientific, Waltham, Massachusetts, United States) and bovine serum albumin (BSA) as a standard.

### 4.10. Statistical Analysis

The effects of NaCl, genotypes, and interaction between them were evaluated at three levels of significance: *p* < 0.05 (*), *p* < 0.01 (**), and *p* < 0.001 (***). Data were subjected to two-way ANOVA and the Fisher’s test was used for mean separation and to provide homogeneous groups for the means (at *p* ≤ 0.01).

All data were process in XLSTAT, a user-friendly statistical software for Microsoft Excel. Statistical elaboration consisted in the production of a correlation matrix (Pearson’s coefficient) to analyze intra-variable links and into a principal component analysis (PCA) to investigate the relationship among different genotypes submitted to different salt stress conditions. The sampling adequacy of individual and set variables was verified by the Kaiser–Meyer–Olkin measure (>0.50) and by Bartlett’s test of sphericity (<0.05). Variables with communality values <0.5 could be removed. PCA was performed and the main components selected by the latent root criterion (eigenvalues >1.0) [22].

A cluster analysis (CA) run after PCA was explained according to correlation matrix. Taken together, multivariate analysis allowed us to identify sensitive cultivars for different NaCl concentration and to identify chemical or physical parameters that better concur to characterize the response of plant to induced salt stress.

## Figures and Tables

**Table 1:** Relative growth rate, node number, shoot number, shoot length (cm), and mean internode length (cm) of olive cv Canino, Sirole, and the transgenic lines Canino AT17-1 and Canino AT17-2 under different concentration of NaCl. Data are represented as mean ± SD. Different letters within the same columns indicating significant differences according to Fisher’s test (*p* < 0.01). Significant effect: * *p* ≤ 0.05; ** *p* ≤ 0.01; *** *p* ≤ 0.001; - not significant.

Genotype	NaCl (mM)	RGR	Node Number	Shoot Number	Shoot Length (cm)	Internode Length (cm)
Canino	0 (Control)	4.57 ± 0.27 ab	2.42 ± 0.44 cd	1.25 ± 0.31 c–f	3.37 ± 0.96 c	1.72 ± 0.74 ab
	50	5.59 ± 1.25 a	1.83 ± 0.41 de	1.08 ± 0.20 d–f	2.72 ± 0.84 c–e	1.81 ± 0.76 ab
	100	4.65 ± 0.79 ab	1.04 ± 0.35 f	1.34 ± 0.25 c–e	2.00 ± 0.49 ef	1.32 ± 0.44 bc
	200	1.58 ± 0.30 fg	0.11 ± 0.17 g	0.12 ± 0.11 g	0.64 ± 0.42 h	0.79 ± 0.24 de
Sirole	0 (Control)	3.88 ± 0.54 a–d	3.54 ± 0.64 a	1.95 ± 0.18 a	6.30 ± 0.68 a	2.16 ± 0.73 a
	50	4.48 ± 1.01 ab	3.21 ± 0.63 ab	1.92 ± 0.30 ab	5.36 ± 0.72 ab	1.74 ± 0.42 ab
	100	2.03 ± 0.19 d–g	1.04 ± 0.47 f	1.46 ± 0.25 cd	2.32 ± 0.55 d–f	1.25 ± 0.53 bc
	200	0.89 ± 0.22 g	-	0 0.12 g	0 0.34 h	-
Canino AT17-1	0 (Control)	3.44 ± 1.02 b–e	2.79 ± 0.39 bc	1.38 ± 0.25 c–e	3.57 ± 0.50 c	1.38 ± 0.27 bc
	50	4.17 ± 0.76 a–c	2.25 ± 0.34 cd	1.17 ± 0.21 c–f	3.04 ± 0.67 cd	1.41 ± 0.29 a–c
	100	3.92 ± 0.26 a–c	1.42 ± 0.48 ef	1.12 ± 0.17 d–f	2.21 ± 0.46 df	1.29 ± 0.36 bc
	200	2.55 ± 0.90 c–g	0.79 ± 0.41 f	0.46 ± 0.28 g	0.73 ± 0.49 gh	0.35 ± 0.28 de
Canino AT17-2	0 (Control)	2.87 ± 1.36 b–f	3.22 ± 0.46 ab	1.54 ± 0.39 bc	4.97 ± 1.10 b	1.79 ± 0.69 ab
	50	3.11 ± 0.51 b–f	2.50 ± 0.42 c	1.00 ± 0.11 ef	3.07 ± 0.58 cd	1.74 ± 0.31 bc
	100	3.72 ± 0.72 b–d	2.65 ± 0.41bc	1.12 ± 0.20 d–f	3.38 ± 0.57 c	1.20 ± 0.24 bc
	200	1.77 ± 0.53 e–g	1.17 ± 0.44 f	0.92 ± 0.19 f	1.63 ± 0.49 fg	0.79 ± 0.30 cd
Source of variation						
Genotype		***	***	***	***	-
NaCl concentration		***	***	***	***	***
Gen × NaCl		**	***	***	***	*

**Table 2:** Olive plantlets of cv Canino and Sirole, and transgenic genotypes Canino AT17-1 and Canino AT17-2 maintained in NaCl-enriched media: (**a**) McKinney index (MKI) and (**b**) tolerance index (TI).

Genotype	NaCl (mM)	MKI ^a^	TI ^b^
Canino	0 (Control)	0	-
	50	0	4.7
	100	2.33	16.5
	200	8.87	23.4
Sirole	0 (Control)	0	-
	50	0.25	9.1
	100	5	18.2
	200	9	20.5
Canino AT17-1	0 (Control)	0	-
	50	0	28.8
	100	0.1	46.4
	200	4.45	47.9
Canino AT17-2	0 (Control)	0	-
	50	0	14.3
	100	0.23	25.9
	200	5.1	32.3

**Table 3:** Kaiser–Meyer–Olkin (KMO) and Bartlett’s test for reduced data.

Kaiser–Meyer–Olkin measure of sampling adequacy (KMO)	0.64
Bartlett’s test of sphericity Approx. (Chi-square)	223.23
Df	66
Sign.	0.001

**Table 4:** Pearson’s correlation matrix. Significant correlation at the 0.05 levels were shown in bold.

Variables	RGR	Node (n)	Shoot (n)	Internode (l)	MKI	Chl a	Chl b	Chla/Chlb	Protein	MDA	Proline	OASTL
RGR	**1**											
Node (n)	**0.57**	**1**										
Shoot (n)	**0.61**	**0.82**	**1**									
Internode (l)	**0.72**	**0.83**	**0.84**	**1**								
MKI	**−0.82**	**−0.86**	**−0.76**	**−0.82**	**1**							
Chl a	**0.51**	0.40	0.24	**0.50**	−0.49	**1**						
Chl b	**0.54**	0.47	0.33	**0.56**	**−0.52**	**0.98**	**1**					
Chla/Chlb	−0.23	**−0.52**	**−0.49**	−0.37	0.30	−0.42	**−0.54**	**1**				
Protein	**−0.58**	**−0.73**	**−0.84**	**−0.81**	**0.69**	−0.18	−0.27	**0.49**	**1**			
MDA	−0.21	−0.15	−0.27	−0.29	0.15	0.31	0.23	0.03	**0.59**	**1**		
Proline	0.13	0.26	0.16	0.11	**−0.47**	0.04	−0.04	0.33	−0.06	0.15	**1**	
OASTL	**−0.60**	**−0.68**	**−0.76**	**−0.87**	**0.69**	**−0.49**	**−0.52**	0.31	**0.85**	**0.33**	−0.17	**1**

**Table 5:** PCA: eigenvalues, explained and cumulate inertia.

	F1	F2	F3	F4	F5	F6	F7	F8	F9	F10	F11	F12
*Initial Eigenvalues*	6.5	2.0	1.5	0.8	0.5	0.4	0.2	0.1	0.0	0.0	0.0	0.0
*Explained Variance (%)*	54.1	16.8	12.4	6.6	3.8	3.1	1.8	0.8	0.4	0.1	0.1	0.0
*Cumulate Inertia (%)*	54.1	**70.9**	83.3	89.9	93.8	96.9	98.7	99.4	99.8	99.9	100.0	100.0

**Table 6:** Electrical conductivity (Ec) expressed in (µS/cm) of the proliferation media enriched with different concentrations of NaCl (50, 100, 200 mM and 0 as control).

Concentration of NaCl	Ec (µS/cm)		
0 mM	7653	±	15
50 mM	13,764	±	12
100 mM	19,905	±	75
200 mM	40,002	±	62.5

**Table 7:** Rating scale consisting of ten classes with the numeric values for the McKinney index (MKI).

Class	Symptoms
0	0 injuries
1	Browning at basal sections of the stem
2	0 to 20% chlorotic symptoms
3	20–50% chlorotic symptoms
4	Up to 10% browning on stem or over 50% chlorotic symptoms
5	10–30% browning of the stem
6	Necrosis on apical leaves
7	30–50% necrosis of the stem
8	50% necrotic symptoms on stem
9	Restricted growth or necrosis on whole stem
10	Necrosis of the whole explant

## Data Availability

The datasets generated during and/or analyzed during the current study are available from the corresponding author on reasonable request.
